# Comparison of Individual Retinal Layer Thicknesses after Epiretinal Membrane Surgery with or without Internal Limiting Membrane Peeling

**DOI:** 10.1155/2018/1256781

**Published:** 2018-10-21

**Authors:** Chul Hee Lee, Min Woo Lee, Eun Young Choi, Suk Ho Byeon, Sung Soo Kim, Hyoung Jun Koh, Sung Chul Lee, Min Kim

**Affiliations:** ^1^Department of Ophthalmology, Institute of Vision Research, Gangnam Severance Hospital, Yonsei University College of Medicine, Seoul, Republic of Korea; ^2^Department of Ophthalmology, Institute of Vision Research, Severance Hospital, Yonsei University College of Medicine, Seoul, Republic of Korea

## Abstract

**Purpose:**

To compare changes in the retinal layer thickness and visual outcomes in patients undergoing epiretinal membrane (ERM) surgery with or without internal limiting membrane (ILM) peeling.

**Methods:**

Seventy-six eyes of 76 patients who underwent ERM surgery from January 2013 to March 2015 at the Department of Ophthalmology, Yonsei University College of Medicine, Seoul, South Korea, were analyzed. While ERM removal with ILM peeling was performed in ILM peeling (P) group (*n*=39), ILM peeling was not performed in non-ILM peeling (NP) group (*n*=37). Retinal layer segmentation was performed using optical coherence tomography images. Individual retinal layer thicknesses before and at 6 months after ERM surgery were compared. The postoperative best-corrected visual acuity (BCVA) was also compared.

**Results:**

In the P group, the thicknesses of retinal nerve fiber layer (RNFL), ganglion cell layer (GCL), and inner plexiform layer (IPL) were significantly reduced. In the NP group, significant decreases in the RNFL, GCL, IPL, inner nuclear layer, and outer plexiform layer were observed. The P group manifested a greater mean postoperative GCL (35.56 ± 1.53 *µ*m vs 29.86 ± 2.16 *µ*m; *p*=0.033) and less loss of GCL (−10.26 ± 1.91 *µ*m vs −19.86 ± 2.74 *µ*m; *p*=0.004) compared to the NP group. No statistically significant differences were observed when comparing the changes in BCVA.

**Conclusions:**

This study demonstrates that ILM peeling for ERM surgery may result in better preservation of GCL compared to no ILM peeling.

## 1. Introduction

The epiretinal membrane (ERM) is an avascular proliferative fibrous tissue composed of extracellular matrix and a polymorphous population of cells, which develops between the vitreous and the internal limiting membrane (ILM). Tangential tractional force on the retina asserted by an ERM leads to distortion of normal retinal structure and layers, causing symptoms such as impairment of central vision, metamorphopsia, macropsia, and monocular diplopia [1, 2]. For many years, the treatment of choice for symptomatic ERMs had been pars plana vitrectomy (PPV) with membranectomy [3]. As ILM peeling has greatly improved the anatomical success rate of macular hole surgery in randomized controlled trials [4, 5], ILM removal has been favored in the treatment of ERM. Although previous studies have described some advantages of ILM peeling for ERM surgery [6, 7], there is still debate over the visual outcomes, safety, and indications for ILM peeling in patients with ERM.

The advantages of ILM removal during ERM surgery include better anatomical outcomes, lower recurrence rates, and better final visual acuity [6–9]. ILM is a transparent structure that defines the boundary between the retina and the vitreous body. It serves as the footplate of Müller cells, astrocytes, and fibroblasts, permitting adhesion and gliosis [10, 11]. ILM removal, therefore, inhibits fibrous membrane formation by removing the scaffold for astrocyte and fibroblast proliferation, which explains its association with lower recurrence and better anatomical success rates. However, ILM peeling during ERM removal may be traumatic to retinal layers resulting in irregularities and indentations on the inner surface of the retina and thinning of the temporal retina [12]. Additionally, some comparative studies have shown that ILM peeling during ERM operations provides no definite functional benefit with respect to improving visual acuity [3, 13].

With the development of automated segmentation of retinal layers using optical coherence tomography (OCT), analysis of changes in individual retinal layers has become possible. A recent study has validated the accuracy of automated segmentation analysis [14, 15]; therefore, segmentation of retinal layers using OCT can be a useful tool for evaluating changes in retinal layers before and after vitreoretinal surgery. Previous studies indicated that preoperative integrity of the inner segment and outer segment line (IS/OS line) [16], preoperative photoreceptor outer segment length [17], and postoperative ganglion cell layer (GCL) thickness [18] are significantly correlated with postoperative best-corrected visual acuity (BCVA). However, there has been no prior comparative analysis of the changes in individual retinal layers by automated segmentation between patients who have undergone ERM surgery with ILM peeling versus without ILM peeling.

The purpose of this study is to analyze the changes in individual retinal layer thickness by automated segmentation in patients who have undergone ERM surgery with or without ILM peeling.

## 2. Methods

### 2.1. Enrollment of Study Population

This was a single-center retrospective study. We analyzed patient records, operative reports, and operation videos of 103 patients (103 eyes) who underwent ERM surgery by two surgeons (MK and SSK) at the Department of Ophthalmology, Yonsei University College of Medicine, Seoul, South Korea, between January 2013 and March 2015. The patients were classified into two groups depending on whether they underwent ILM peeling: ILM peeling (P) group with PPV plus epiretinal membranectomy plus ILM peeling and non-ILM peeling (NP) group with PPV plus epiretinal membranectomy. Only patients diagnosed with idiopathic ERM were included. Patients with other combined forms of maculopathy, such as macular hole, lamellar macular hole, diabetic macular edema, or retinal vein occlusion were excluded. Patients were also excluded from the analysis if they required reoperation or intravitreal injections within the 1-year follow-up period to treat postoperative complications such as retinal detachment, dislocation of intraocular lens, pseudophakic cystoid macular edema, and choroidal neovascularization. Only those patients who did not show significant posterior capsular opacity after the ERM surgery were included in the study. This study was approved by the institutional review board of Yonsei University College of Medicine (IRB approval number: 3-2016-0278) and was conducted in accordance with the tenets of the Declaration of Helsinki.

### 2.2. Preoperative Examination and Automated Segmentation

All past medical history and preoperative ophthalmologic data for each patient were reviewed. Results of the following preoperative evaluations were recorded: BCVA obtained by the Snellen visual acuity chart, which was converted to a logarithm of the minimum angle of resolution (logMAR) value for statistical analysis; slit-lamp biomicroscopy; intraocular pressure, as determined using a noncontact tonometer; color fundus photography; biometry measurements, obtained by the ZEISS IOLMaster® 500 (Carl Zeiss AG; Heidenheim, Germany); and OCT images, taken by the spectral domain OCT (SD-OCT; Spectralis®; Heidelberg Engineering, Heidelberg, Germany).

**Figure 1:**
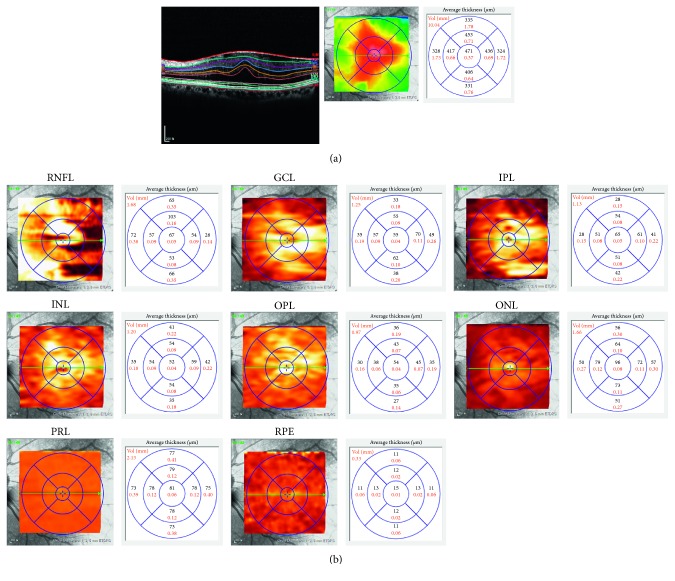
An example of automated retinal layer segmentation performed preoperatively on a patient with an epiretinal membrane. (a) Automated segmentation of retinal layers was performed by the built-in software of spectral domain OCT (SD-OCT; Spectralis®; Heidelberg Engineering, Heidelberg, Germany). (b) The segmentation analysis was performed within a 6-mm diameter circle centered on the fovea, as defined in the Early Treatment of Diabetic Retinopathy Study (ETDRS). The average retinal thickness in each of the 8 macular sectors was automatically calculated: retinal nerve fiber layer (RNFL), ganglion cell layer (GCL), inner plexiform layer (IPL), inner nuclear layer (INL), outer plexiform layer (OPL), outer nuclear layer (ONL), photoreceptor layer (PRL), and retinal pigment epithelium (RPE). The average retinal layer thickness at each macular sector was calculated at the 1-mm center circle, 3-mm inner ring, and 6-mm outer ring of the ETDRS.

Automated segmentation of retinal layers was performed by the built-in software, which automatically calculated the average retinal thickness in each of the individual retinal layers: retinal nerve fiber layer (RNFL), GCL, inner plexiform layer (IPL), inner nuclear layer (INL), outer plexiform layer (OPL), outer nuclear layer (ONL), photoreceptor layer (PRL), and retinal pigment epithelium (RPE). The segmentation analysis was performed by two independent observers (CHL and EYC). Analysis was performed within a 6-mm diameter circle centered on the fovea, as defined in the Early Treatment of Diabetic Retinopathy Study (ETDRS) [19]. The diameters of the central circle, inner ring, and outer ring were 1 mm, 3 mm, and 6 mm, respectively (Figure 1).

### 2.3. Surgical Technique

For all patients, a 25-gauge PPV was performed (CONSTELLATION® Vision System, Alcon, Fort Worth, TX, USA). After performing core vitrectomy, triamcinolone was injected intravitreally to better visualize the vitreous gel and ERM. After removing the detached vitreous gel and the posterior hyaloid membrane, removal of the ERM was performed using intraocular forceps.

**Figure 2:**
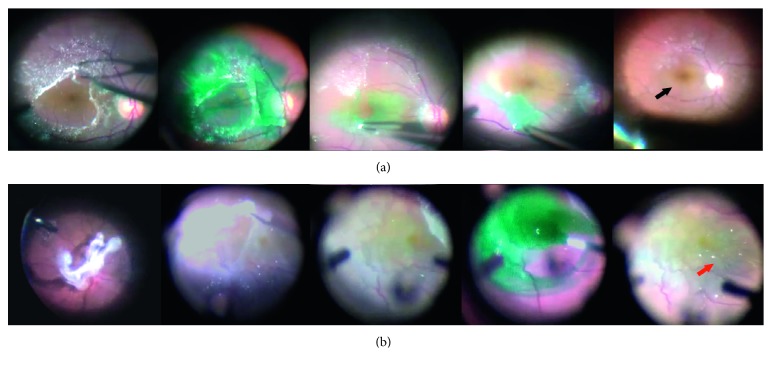
Examples of epiretinal membrane surgery with and without internal limiting membrane peeling. (a) For the ILM peeling (P) group, initial removal of posterior hyaloid membrane and ERM was performed using intraocular forceps with assistance of triamcinolone injection for better visualization. The ILM was double stained with 0.2 mL of 1 mg/mL indocyanine green (ICG) solution (DID-Indocyanine Green inj, Dongindang Pharmaceutical, Siheung, Republic of Korea). The ILM, which was stained light-green, was peeled using a 25-gauge ILM forceps. An area of approximately 2 to 3 disc diameters centered on the macula of the ILM was peeled. After the initial ILM peeling, ICG dye solution was reinjected to visualize residual ILM. There was no residual ILM visible by ICG dye staining within 2 to 3 disc diameters of the macular center (black arrow). (b) For the non-ILM peeling (NP) group, after initial posterior hyaloid membrane and ERM removal, ICG dye solution was injected over the macula region to ensure that ILM remained intact (red arrow).

In the P group, the ILM was stained with 0.2 mL of 1 mg/mL indocyanine green (ICG) solution (DID-Indocyanine Green inj, Dongindang Pharmaceutical, Siheung, Republic of Korea). Both surgeons used the same concentration of ICG dye. After injecting the 1 mg/mL ICG solution at the macula area, the infusion was turned on immediately followed by aspiration of ICG dye with the vitrectomy cutter for minimal ICG dye circulation within the vitreous cavity. The ILM was peeled of an area of approximately 2 to 3 disc diameters centered on the macula using a 25-gauge ILM forceps. After the initial ILM peeling, ICG dye solution was reinjected to visualize residual ILM. Residual ILM was peeled until there was no ILM visible by ICG dye staining within 2 to 3 disc diameters of the macular center (Figure 2(a)).

In the NP group, ICG dye solution was injected over the macula region after epiretinal membranectomy to ensure that ILM remained intact. Patients with ILM unstained after simple membranectomy were excluded from the NP group (Figure 2(b)).

### 2.4. Postoperative Examination

To determine the effects of ILM peeling on BCVA and anatomical structure of the retinal layers, the BCVA and automated segmentation analysis of SD-OCT at 6 months after the operation were analyzed. The change in retinal layer thickness was determined by subtracting the preoperative retinal layer thickness from the postoperative retinal layer thickness at the 1 mm central circle. The change in BCVA was calculated by subtracting the preoperative BCVA from the postoperative BCVA at 6 months follow-up.

### 2.5. Statistical Analyses

For all segmentation data, only the retinal layer thicknesses in the central circle at 1 mm were compared. The mean age and preoperative BCVA, biometry data, and segmentation data of the two groups (P group and NP group) were compared using independent Student's *t*-tests. The mean postoperative BCVA and segmentation data of the two groups were also compared using independent Student's *t*-tests. Within each group, the significance of the change in thickness of each retinal layer from before surgery to 6 months after surgery was determined by paired sample *t*-tests. The correlation between the thickness of each layer and postoperative BCVA was calculated by Pearson's correlation coefficient. A value of *p* < 0.05 was accepted as statistically significant.

Interrater agreement between the two observers was analyzed for all segmentation data by calculating intraclass correlation coefficients (ICCs). All statistical analyses of the data were performed using IBM SPSS 23.0 software for Windows (SPSS/IBM Corporation, Chicago, IL, USA). Data are presented as mean ± standard deviation, except where indicated otherwise.

## 3. Results

### 3.1. Baseline Characteristics

Out of the 103 patients who underwent ERM surgery, 76 patients (76 eyes) with clinically confirmed idiopathic ERM satisfied the inclusion criteria and were included in the final analysis (P group *n*=39, NP group *n*=37). Sample size calculation was done by using the modified Cochran's formula. By using this formula, the sample size of 76 eyes met 95% confidence level with 6% margin of error about the population of 103 cases that underwent ERM surgery by two surgeons (MK and SSK) at the Department of Ophthalmology, Yonsei University College of Medicine between January 2013 and March 2015. There were no significant differences in patient age, BCVA, axial length, and spherical equivalent diopter between the two groups (Table 1). In addition, the mean preoperative segmented retinal layer thicknesses at each macular sector did not exhibit any significant differences (Table 1). Simultaneous cataract surgery was performed for all phakic eyes (P group: 61.5%; NP group: 62.2%; independent Student's *t*-tests, *p*=0.999), and posterior capsular opacities were removed in all pseudophakic patients (P group: 38.5%; NP group: 37.8%; *p*=0.999). In the P group, the average ILM removal time was 2.4 ± 0.5 minutes for surgeon 1 (MK) and 2.3 ± 0.7 for surgeon 2 (SSK) (*p*=0.999). In the NP group, the average ERM removal time was 2.2 ± 0.2 minutes for surgeon 1 (MK) and 2.2 ± 0.4 minutes for surgeon 2 (SSK) (*p*=0.999).

### 3.2. Individual Retinal Layer Segmentation and BCVA at 6 Months Postoperatively

At 6 months postoperatively, the mean GCL thickness was significantly higher in the P group than in the NP group (P group: 35.56 ± 1.53 *µ*m; NP group: 29.86 ± 2.16 *µ*m; *p*=0.033; Table 2). There was no significant difference in BCVA between the two groups (P group: 0.11 ± 0.02; NP group: 0.16 ± 0.02; *p*=0.099; Table 2). No significant correlation between postoperative GCL and postoperative BCVA was observed in both groups (P group: Pearson *r*=0.218, *p*=0.182; NP group: Pearson *r*=0.049, *p*=0.775).

In the analysis of mean differences in retinal layer thickness before and after 6 months operation, the P group exhibited less loss of GCL thickness when compared to the NP group (P group: −10.26 ± 1.91 *µ*m; NP group: −19.86 ± 2.74 *µ*m; *p*=0.004; Table 3). The mean change in thickness in all other segmented layers showed no significant differences (Table 3).

In paired *t*-test analysis, the P group showed significant reduction in the RNFL, GCL, and IPL thicknesses at 6 months after surgery. On the other hand, significant decreases in thickness that extended into the deeper layers, including the RNFL, GCL, IPL, INL, and OPL, were observed in the NP group (Table 3). The BCVA of both groups improved significantly after surgery (P group: *p* < 0.0001; NP group: *p*=0.006; paired *t*-tests).

The ICCs for the preoperative and postoperative segmentation data indicated excellent interrater agreement in all layers.

## 4. Discussion

ILM peeling for ERM surgery resulted in less loss of GCL thickness compared to no ILM peeling.

A novel finding of this study is that the P group exhibited significantly lower reduction of GCL thickness compared to the NP group. This contradicts many previous concerns regarding iatrogenic trauma and retinal toxicity produced by ICG dye guided ILM peeling.

In previous studies using electron microscopy, findings indicated possible Müller cell damage caused by the ILM peeling procedure [20, 21]. However, these peeled ILM samples only contained Müller cells and myofibroblasts and were void of ganglion cells, photoreceptors, or RPE cells [20]. Another recent study showed that specimens acquired from ILM abrasion using a tano diamond-dusted membrane scraper did not contain RNFL or neuronal cells that lay beneath the ILM [22]. In accordance with our results, these studies suggest that iatrogenic trauma may be confined to Müller cells, and other neuronal cells are minimally affected by the procedure.

Unfortunately, we could not perform auto fluorescence, microperimetry, or visual field testing for evaluating ICG dye toxicity in terms of RPE cell function. However, our study shows that the use of ICG dye with 1 mg/mL concentration during ERM surgery does not induce significant retinal toxicity, in terms of preserving retinal thickness, including the RPE layer. In agreement with our results, Kwok et al. have demonstrated that there was no clinically significant ICG toxicity after ILM peeling angiographically [23]. There have been some case reports of poor visual outcomes due to ICG dye toxicity after successful macular hole closure [24]. However, with a macular hole, the RPE and other retinal layers at the fovea are directly exposed to the vitreous cavity, whereas in the presence of an ERM, these layers are enclosed by the fibrotic membrane and ILM. We speculate that the risk of foveal exposure to ICG dye would be lower in patients with an ERM.

The reason for the relative preservation of postoperative GCL in the P group is unclear. However, we hypothesize that induction of Müller cell injury during ILM peeling may have triggered reactive gliosis, resulting in subsequent thickening of GCL compared to the NP group. On the retinal side of the ILM obtained after ERM surgery, electron micrographs revealed segments of Müller cell footplates in ILM specimens, which shows that ILM peeling generates Müller cell injury [21]. In addition, injured Müller cells have a role in retinal neural regeneration and repair as described in previous studies performed on rodent and human retinal tissues [25–28]. Hypothetically, ILM peeling, having induced Müller cell injury, may have activated reactive gliosis at the GCL level with the RNFL serving as Müller cell footplate.

However, it is unclear whether greater GCL thickness as shown by our study necessarily means a recovery of healthy neuronal cells. Previous studies have shown decreased retinal function on multifocal electroretinogram and visual field sensitivity after ILM peeling [21, 29]. Our study showed that there was no correlation between postoperative GCL thickness and postoperative BCVA in the P group (Pearson *r*=0.218, *p*=0.182). The relative preservation of GCL after ILM peeling may be a result of a reactive gliosis after initial injury on Müller cells, rather than a healthy regeneration of ganglion cells. Further study about the changes that occur at cellular level after ILM peeling is required to clarify these results.

There is no significant difference in postoperative BCVA between ILM peeling and no ILM peeling for ERM surgery.

Both groups exhibited significant improvements in BCVA after ERM surgery. However, ILM peeling did not result in superior visual outcomes regarding central visual acuity. Our results agree with a recent randomized controlled study that compared the BCVA of ILM peeling and no ILM peeling [30]. In other studies, some have reported superior outcomes in ILM peeling group, while some have reported opposing results [6, 7, 31]. However, the advantage of our study over these previous studies is that the proportion of eyes with and without ILM peeling was similar (P group: 51.3% versus NP group: 48.7%), which adds representativeness and objectivity to our data.

Our study has a few limitations. First, although the surgical protocols in the two groups were identical except for ILM peeling, two surgeons performed operations. However, there was no significant difference between the two surgeons in operation time, ERM removal time, or ILM removal time. Also, since there were a sufficient and approximately equal number of each surgeon's patients in both groups, the surgeon factors may have been minimized. Second, epiretinal membranectomy without ILM peeling does not necessarily result in complete preservation of the ILM, as ILM could be removed along with the ERM during the membrane removal procedure. Unfortunately, we could not perform a histological study proving that the ILM was completely preserved after ERM removal in the NP Group. As an alternative to a histological study, we have done the best we could clinically by thoroughly reviewing our surgical videos to include only those eyes that showed complete peeling of ILM in the P group and cases with ILM as completely preserved as possible in the NP Group grossly (Figure 2). Third, there are insufficient data about the changes that occur at cellular levels after surgical manipulation of the ILM, a key finding to explain our data.

In conclusion, this study demonstrates a novel finding that ILM peeling during ERM surgery may result in better preservation of GCL compared to ERM surgery without ILM peeling. We cautiously speculate that the removal of ILM and subsequent Müller cell injury may have induced reactive gliosis. Future studies regarding the changes inflicted on Müller cells after ILM removal in the human retina are required to support our results and confirm our findings.

## Figures and Tables

**Table 1:** Baseline characteristics and preoperative automated retinal layer segmentation.

	P group (ILM peeling) (*n*=39) Mean ± SD	NP group (non-ILM peeling) (*n*=37) Mean ± SD	*p* value
Age (years)	66.59 ± 1.41	68.73 ± 1.14	0.245
Preoperative BCVA (logMAR)	0.23 ± 0.03	0.27 ± 0.03	0.255
Spherical equivalent (D)	0.41 ± 0.36	0.43 ± 0.31	0.958
Axial length (mm)	23.72 ± 0.20	23.31 ± 0.18	0.135
Total retinal thickness (*µ*m)	466.4 ± 11.31	458.7 ± 10.25	0.616
RNFL thickness (*µ*m)	84.46 ± 11.79	70.78 ± 9.81	0.378
GCL thickness (*µ*m)	45.82 ± 1.39	49.73 ± 1.97	0.106
IPL thickness (*µ*m)	45.97 ± 1.76	46.43 ± 1.86	0.858
INL thickness (*µ*m)	50.49 ± 1.70	52.38 ± 1.71	0.435
OPL thickness (*µ*m)	37.82 ± 1.32	40.46 ± 1.68	0.217
ONL thickness (*µ*m)	114.2 ± 4.60	111.4 ± 4.35	0.660
PRL thickness (*µ*m)	71.56 ± 0.69	70.84 ± 0.64	0.444
RPE thickness (*µ*m)	16.21 ± 0.47	16.84 ± 0.52	0.369

BCVA = best-corrected visual acuity; SD = standard deviation; ILM = internal limiting membrane; RNFL = retinal nerve fiber layer; GCL = ganglion cell layer; IPL = inner plexiform layer; INL = inner nuclear layer; OPL = outer plexiform layer; ONL = outer nuclear layer; PRL = photoreceptor layer; RPE = retinal pigment epithelium. Independent Student's *t*-test for statistical analysis between Group 1 and Group 2 for retinal layers, BCVA, age, spherical equivalent, and axial length.

**Table 2:** Automated retinal layer segmentation and best-corrected visual acuity at 6 months after epiretinal membrane surgery.

	P group (ILM peeling) (*n*=39) Mean ± SD	NP group (non-ILM peeling) (*n*=37) Mean ± SD	*p* value
Total retinal thickness (*µ*m)	378.9 ± 5.89	360.8 ± 8.94	0.091
RNFL thickness (*µ*m)	21.67 ± 1.47	23.95 ± 1.80	0.327
*GCL thickness (µm)*	35.56 ± 1.53	29.86 ± 2.16	0.033^*∗*^
IPL thickness (*µ*m)	34.05 ± 1.17	31.41 ± 1.81	0.219
INL thickness (*µ*m)	45.46 ± 1.55	44.49 ± 2.45	0.735
OPL thickness (*µ*m)	35.05 ± 1.16	33.30 ± 1.49	0.353
ONL thickness (*µ*m)	117.5 ± 3.90	109.5 ± 2.98	0.112
PRL thickness (*µ*m)	72.77 ± 0.72	72.05 ± 0.67	0.470
RPE thickness (*µ*m)	17.13 ± 0.93	17.24 ± 0.80	0.926
BCVA (logMAR)	0.11 ± 0.02	0.16 ± 0.02	0.099

BCVA = best-corrected visual acuity; SD = standard deviation; ILM = internal limiting membrane; RNFL = retinal nerve fiber layer; GCL = ganglion cell layer; IPL = inner plexiform layer; INL = inner nuclear layer; OPL = outer plexiform layer; ONL = outer nuclear layer; PRL = photoreceptor layer; RPE = retinal pigment epithelium. Independent Student's *t*-test for statistical analysis between Group 1 and Group 2 for retinal layers and BCVA.

**Table 3:** Difference in segmented retinal layer thicknesses and best-corrected visual acuity before and at 6 months after epiretinal membrane surgery.

Difference	P group (ILM peeling)	*p* value (preop vs POD 6 month)	NP group (non-ILM peeling)	*p* value (preop vs POD 6 month)	*p* value (P group vs NP group)
Total retinal thickness (*µ*m)	−87.51 ± 9.87	<0.0001^†^	−97.95 ± 8.35	<0.0001^†^	0.425
RNFL thickness (*µ*m)	−62.79 ± 11.43	<0.0001^†^	−46.84 ± 9.21	<0.0001^†^	0.283
GCL thickness (*µ*m)	−10.26 ± 1.91	<0.0001^†^	−19.86 ± 2.74	<0.0001^†^	0.004^*∗∗*^
IPL thickness (*µ*m)	−11.92 ± 1.89	<0.0001^†^	−15.03 ± 2.37	<0.0001^†^	0.306
INL thickness (*µ*m)	−5.03 ± 2.49	0.050	−7.89 ± 3.29	0.022^*∗*^	0.486
OPL thickness (*µ*m)	−2.77 ± 1.71	0.114	−7.16 ± 1.58	0.002^*∗∗*^	0.064
ONL thickness (*µ*m)	3.26 ± 5.29	0.542	−1.89 ± 4.88	0.721	0.478
PRL thickness (*µ*m)	1.21 ± 0.77	0.126	1.22 ± 0.82	0.194	0.992
RPE thickness (*µ*m)	0.92 ± 0.93	0.326	0.41 ± 0.78	0.672	0.672
BCVA (logMAR)	−0.11 ± 0.02	<0.0001^†^	−0.11 ± 0.03	0.006^*∗∗*^	0.950

BCVA = best-corrected visual acuity; POD = postoperative day; SD = standard deviation; ILM = internal limiting membrane; RNFL = retinal nerve fiber layer; GCL = ganglion cell layer; IPL = inner plexiform layer; INL = inner nuclear layer; OPL = outer plexiform layer; ONL = outer nuclear layer; PRL = photoreceptor layer; RPE = retinal pigment epithelium. Independent Student's *t*-test for statistical analysis between Group 1 and Group 2 for difference of retinal layers and BCVA: ^*∗*^*p* < 0.05, ^*∗∗*^*p* < 0.01, ^†^*p* < 0.001. Paired sample *t*-test within Group 1 and Group 2 for statistical analysis: ^*∗*^*p* < 0.05, ^*∗∗*^*p* < 0.01, ^†^*p* < 0.001.

## Data Availability

The datasets used and/or analyzed during the current study are available from the corresponding author on reasonable request. All data generated or analyzed during this study are included in this article.
